# Retrospective analysis of neoplasms in patients using angiotensin receptor blockers

**DOI:** 10.1038/s41598-024-64867-y

**Published:** 2024-07-09

**Authors:** Arvind Kumar Sharma, Shruti Rastogi, Ramesh K. Goyal

**Affiliations:** 1 Delhi Pharmaceutical Sciences and Research University, Pushp vihar Sector 3, New Delhi, 110017 India; 2 Indian Pharmacopoeia Commission, Ministry of Health & Family Welfare, Govt. of India, Sector-23, Raj Nagar, Ghaziabad, 201002 Uttar Pradesh India

**Keywords:** Adverse drug reaction, EudraVigilance, Pharmacovigilance, Angiotensin receptor blockers, Hypertension, Cardiology, Health care, Medical research, Signs and symptoms

## Abstract

In recent years, regulatory agencies have raised concerns about the presence of potentially carcinogenic substances in certain formulations of Angiotensin Receptor Blockers (ARBs). Specifically, nitrosamines and azido compounds have been identified in some ARB products. Nitrosamines are known to have carcinogenic properties and are associated with an increased risk of neoplasms. Spontaneous safety reports from the EudraVigilance Data Analysis System (EVDAS) database were analyzed to investigate cases of neoplasms associated with ARBs. A disproportionality analysis was conducted, calculating the reporting odds ratio (ROR) and 95% confidence intervals (CIs) using a case/non-case approach for each ARB drug. The EVDAS database contained 68,522 safety reports related to ARBs (including Azilsartan, Candesartan, Irbesartan, Olmesartan, Losartan, Valsartan, and Telmisartan), among which 3,396 (5%) cases were associated with neoplasms. The majority of these cases were reported in Germany (11.9%), followed by France (9.7%). Approximately 70% of the reports were submitted by healthcare professionals such as physicians and nurses. Among the ARBs, valsartan had the highest ROR for neoplasm (ROR 1.949, 95% CI 1.857–2.046). This association remained significant when comparing ARBs with other classes of antihypertensive drugs, including ACE inhibitors, beta-blockers, calcium channel blockers, and diuretics. Our study identifies a possible signal of an association between ARBs, particularly valsartan, and the risk of neoplasms. However, further observational and analytical studies are necessary to confirm these findings and elucidate the underlying mechanisms.

## Introduction

According to epidemiologic research, hypertension (HTN) is the most frequent disease worldwide, accounting for more than 10 crore deaths and almost 20.8 crores disability adjusted life years (DALY) worldwide^1,2^. In the previous 30 years, the incidence of HTN in individuals in age-group between 30 and 79 years has increased from 65 to 128 crores^3^. The HTN treatment guidelines recommend normal blood pressure (BP) as less than 120/80 mm Hg; elevated as 120–129/less than 80 mm Hg; HTN stage 1 as 130–139- or 80–89-mm Hg and HTN stage 2 as more than equal to 140 or more than equal to 90 mm Hg^4^. The growing prevalence of HTN is essentially consistent worldwide, regardless of socioeconomic position, i.e., in poorer, middle, and higher-income countries. HTN becomes more frequent with age, reaching a prevalence of more than 60% in adults over the age of 60. Successful management of HTN is the key in minimizing the disease burden and to increase the longevity in the population^2^.

The link between BP reduction and the prevention of cardiovascular disease is widely recognized^5^. Thus, the need for management of HTN arises which basically involves lifestyle changes, dietary sodium restrictions, moderation of alcohol consumption, weight management, physical fitness, smoking cessation and pharmacological therapy^6^. The drugs recommended for the treatment of HTN are Angiotensin Receptor Blockers (ARBs), Angiotensin Converting Enzyme (ACE) inhibitors, Beta-blockers (BB), Calcium Channel Blockers (CCBs) and Diuretics^7^.

ARBs are drugs that block type 1, angiotensin II receptor (AT_1_R)^8^ by displacing angiotensin II from AT_1_R site and increasing the stimulation of type 2, angiotensin II receptor (AT_2_R). AT_1_R is an octapeptide hormone that causes vasoconstriction, fluid and sodium retention, myocyte and smooth muscle cell hypertrophy, and is known to raise BP or cause other cardiovascular illnesses^9^. ARBs are known to treat HTN but are also beneficial in patient’s intolerant to ACE inhibitors and are effective in treatment of HTN, heart failure, diabetic nephropathy and prevention of other related cardiovascular diseases^10^. Approximately 25% of hypertensive patients, accounting for nearly 200 million patients are taking ARBs globally^8^. Currently, nine ARBs mainly indicated to treat HTN are marketed worldwide- azilsartan, candesartan, irbesartan, olmesartan, losartan, valsartan, telmisartan, fimasartan and eprosartan. Fimasartan a recently approved drug has gained approval in Korea, China, India, Singapore and Russia^11^.

In July 2018, the USFDA regulators identified that several formulations of ARBs contain N-nitrosodimethylamine (NDMA), a potential human carcinogen and recalled commonly used ARB (valsartan). FDA estimated that if 8000 patients received highest dose of valsartan once daily for consecutively 4 years, then additional one case of neoplasm may occur from the recalled batches^12^.

The potential biological mechanisms linking ARB usage to an increased risk of neoplasms include several pathways. ARBs block the AT_1_R), resulting in increased activation of the AT_2_R, which can promote cell proliferation and angiogenesis, aiding tumor growth. Normally, angiotensin II induces apoptosis via AT_1_R, but blocking this receptor may reduce apoptosis, allowing potentially cancerous cells to survive^13^. Additionally, ARBs can influence the expression of vascular endothelial growth factor (VEGF), a crucial factor in angiogenesis. Enhanced angiogenesis supports tumor growth by improving blood supply to neoplastic cells^14^. ARBs also alter immune responses and inflammatory pathways; chronic inflammation is a known risk factor for neoplasms as it can lead to DNA damage and create a tumor-friendly microenvironment^15^. Moreover, ARBs may affect the balance of reactive oxygen species (ROS), leading to DNA damage and mutations, which contribute to cancer development. Some ARBs have been shown to increase oxidative stress, further promoting carcinogenesis^16^. They might also interfere with tumor suppressor pathways, such as the p53 pathway, diminishing the body's ability to suppress abnormal cell growth^17^. Additionally, ARBs can affect insulin sensitivity and glucose metabolism, with dysregulation of these metabolic pathways being associated with increased cancer risk^18^. While these mechanisms suggest a plausible link between ARBs usage and increased neoplasm risk, the evidence remains inconclusive.

A meta-analysis published in year 2010 observed that those patients who were exposed to ARBs resulted in a considerably elevated risk of neoplasm when compared to placebo group. i.e., 7.2% versus 6.0%; (Risk ratio 5%; 95% CI 1.01–1.15; p = 0.016)^19^. Since, then many trials have been performed showing heterogenous results, with some suggesting increased neoplasm risk with ARBs^20,21^ and other no risk with ARBs^22–24^. Given the current increase in recalls of multiple ARB drugs containing prescription products due to a potentially neoplasm-causing impurity, a thorough investigation of the causes behind these contradicting conclusions is necessary. Aggregate exposure is a significant factor in the epidemiology of chronic diseases, including neoplasm. Regrettably, neither regulatory agency studies^25^ nor additional analyses of randomized trials^26–28^ have investigated the link between aggregate exposure to ARBs and risk of neoplasm. As a result, the current study is designed to investigate the link between the exposure and risk associated with use of ARBs using spontaneously reported individual case safety reports (ICSRs) to EudraVigilance database in order to shed more light on the link between ARBs and neoplasm and to determine if different levels of continuous ARBs exposure can account for the heterogeneity observed in these trials.

## Material and methods

**Figure 1:**
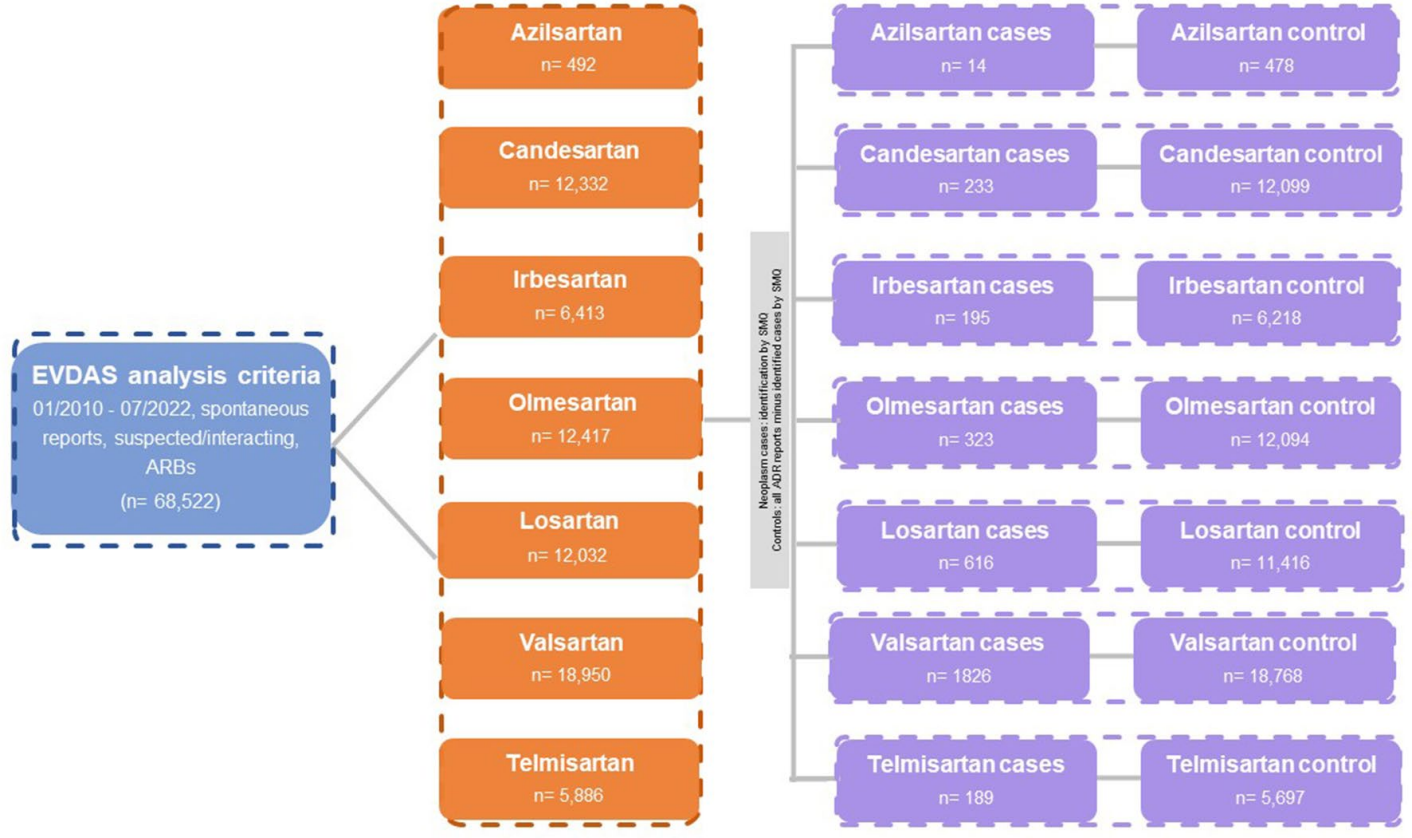
Spontaneous ADR reports of angiotensin receptor blockers from EudraVigilance database.

This is a retrospective, observational, pharmacovigilance study performed using EudraVigilance data analysis system (EVDAS) database. The spontaneous ADR reports received between January 1, 2010 and July 7, 2022 were screened in which either of the ARBs “Azilsartan”, “Candesartan”, “Irbesartan”, “Olmesartan”, “Losartan”, “Valsartan” and “Telmisartan” were reported as ‘suspected/interacting’ drug mono substance (query date: 30/07/2022) (Fig. 1).

The potential confounders in this analysis were addressed through descriptive analyses that detailed the baseline characteristics of the study population, including age, sex, comorbidities, and other relevant variables. The authors gathered clinical information on different ARBs and the countries reporting each ICSRs. Additionally, the outcome of the adverse reaction (classified as serious or not serious), the region, and the reporter's qualifications were documented.

An Adverse Drug Reaction (ADR) is defined as serious if it results in death, is life-threatening, necessitates or prolongs hospitalization, results in significant disability, or is a congenital anomaly/birth defect. This classification of seriousness differs from the clinical severity of the ADR.

Neoplasm cases for each ARB were identified using the standardized MedDRA Query (SMQ) for “malignancies (narrow)”^29^. A case/non-case analysis was conducted to assess the association between the studied ARBs and neoplasms. Cases were identified using the preferred term “Neoplasms benign, malignant, and unspecified (including cysts and polyps).” Non-cases (controls) included all other ADR reports recorded in the EVDAS during the same period. Exposure to the researched ARBs was defined as exposure in both cases and non-cases.

The reported odds ratio (RORs) and their 95% confidence intervals (CIs) between a drug and a specific adverse drug response were estimated as a disproportionality measurement using case/non-case analysis. A 2 × 2 table was used to calculate the ROR using the formula, ROR = ad/cb (where a = exposed cases; b = exposed non-cases; c = nonexposed cases; and d = nonexposed non-cases). We chose ROR among the various disproportionality approaches because it is the one recommended by EVDAS^30^. Furthermore, Rothman et al.^31^ concluded that ROR might be used to determine the relative risk for ADR database, if spontaneous reporting is taken into account as a data source for a case–control study. Because ROR allows for the use of multivariate logistic regression, confounding and interaction effects can be considered^32^. According to European Medicines Agency, a drug-event combination is a signal of disproportionate reporting when the following conditions are met: the lower bound of the 95% CI of the ROR is more than 1 and the number of individual cases is more than or equal to 3.

In addition, comparisons were made between the ARB drugs Azilsartan, Candesartan, Irbesartan, Olmesartan, Losartan, Valsartan, and Telmisartan and their respective controls, as well as between ARB neoplasm cases and all other antihypertensive drugs, including ACE inhibitors, BBs, CCBs, and diuretics. GraphPad Prism 6.0 (GraphPad Software, Inc., San Diego, CA) was used for all statistical analyses.

### Ethics approval

The research was performed in accordance with relevant guidelines/regulations.

## Results

In EDVAS, during the study period, a total of 4,464,148 spontaneous cases of adverse reactions were reported. Of these, 232,506 (5.20%) were of neoplasm. ARBs were involved in 68,522 spontaneous cases (azilsartan, n = 492; candesartan, n = 12,322; irbesartan, n = 6413; olmesartan, n = 12,417; losartan, n = 12,032; valsartan, n = 18,950 and telmisartan, n = 5886) among which 3396 (5%) cases were reported of as “suspected” of neoplasm (azilsartan, n = 14; candesartan, n = 233; irbesartan, n = 195; olmesartan, n = 323; losartan, n = 616; valsartan, n = 1826 and telmisartan, n = 189). Of the cases of neoplasm reported 1568 (46.1%) were females.

For general cases of ARBs the major cases were reported in the Germany (n = 8180; 11.9%), followed by France (n = 6657; 9.7%). Approximately 70% of the cases were reported by healthcare professionals including physicians, nurses etc. As per European Union (EU), 80% cases were serious and 99% cases of neoplasm were serious criteria. Table 1 summarizes the general characteristics of the cases. The neoplasm was observed in 53.7% patients treated with Valsartan and 18% of patients treated with Losartan for the specific characteristic cases of “Neoplasms benign, malignant, and unspecified (incl cysts and polyps)”. Non-healthcare professionals, including patients and marketing authorization holders (MAH), reported around 55.5% of neoplasm instances. And 83% of the instances were recorded in nations outside the European Economic Area (EEA). Table 2 summarizes the main characteristics of cases of benign, malignant, and unspecified neoplasms (including cysts and polyps).

**Table 1:** General characteristics of ARBs in EudraVigilance.

	Azilsartan (n = 492) %	Candesartan (n = 12,332) %	Irbesartan (n = 6413) %	Olmesartan (n = 12,417) %	Losartan (n = 12,032) %	Valsartan (n = 18,950) %	Telmisartan (n = 5886) %
Sex (n, %)							
Female	49.2%	56.2%	53.8%	55.7%	55.1%	52.6%	52.9%
Male	45.7%	40.6%	42.9%	41.8%	40.1%	40.4%	42.4%
Not specified	5%	3.2%	3.3%	2.4%	4.9%	7%	4.7%
Age group							
0–18 years	0%	1.15%	0.49%	0.2%	1.1%	0.7%	0.4%
18–64 years	29.7%	31.5%	30.1%	47.3%	31.5%	28.3%	33.9%
65–85 years	40.9%	40.5%	46.2%	40.6%	41.1%	39.8%	40.7%
More than 85 years	8.5%	7.4%	9.2%	4.9%	6.2%	7.1%	7%
Not specified	22.9%	19.5%	14%	6.9%	20.1%	24.2%	18%
Outcome (n, %)							
Serious	92%	65.6%	74.9%	92.1%	81.1%	87.2%	73.6%
Non serious	7.9%	33.5%	24.5%	7.9%	18%	12.3%	26%
Not specified	0%	0.75%	0.6%	0.1%	1%	0.5%	0.3%
Region (n, %)							
EEA	10%	64.2%	62.7%	22.9%	40.6%	34.8%	40.9%
Non-EEA	90%	40.6%	37.3%	77.1%	59.3%	65.2%	59.1%
Not specified	0	0	0	0	0	0	0
Country specific							
Germany	4.8%	27.6%	7.6%	4%	5.5%	12.8%	11.4%
Greece	1.4%	0.17%	2.0%	0.8%	0.1%	0.5%	0.5%
Portugal	2.8%	0.24%	0.8%	0.8%	0.6%	0.3%	0.6%
France	0	12.4%	29.6%	4.8%	4.6%	8.3%	8.3%
United Kingdom	0	5.0%	4.3%	0.9%	6.7%	1.9%	0.9%
Sweden	0	4.9%	0.71%	0%	4.5%	0.3%	0.3%
Netherlands	0	4.8%	6.8%	0.5%	5.8%	2.3%	2.8%
Reporter qualification							
HCP	77.2%	69.5%	75.7%	73.2%	74.1%	61.6%	75.7%
Non-HCP	22.7%	30%	23.6%	26.7%	25.2%	37.5%	24%
Not specified	0	0.5%	0.7%	0.2%	0.8%	0.9%	0.3%

**Table 2:** General characteristics of cases of “Neoplasms benign, malignant and unspecified (incl cysts and polyps)” associated with ARBs in EudraVigilance.

	Azilsartan (n = 14) %	Candesartan (n = 233) %	Irbesartan (n = 195) %	Olmesartan (n = 323) %	Losartan (n = 616) %	Valsartan (n = 1826) %	Telmisartan (n = 189) %	Total (n = 3396) %
Sex (n, %)								
Female	50%	55.8%	41%	59.1%	57.6%	38.6%	53.4%	46.1%
Male	50%	41.2%	49.7%	38.1%	34.6%	39.5%	40.7%	39.2%
Not specified	0	3%	9.2%	2.1%	8.1%	22%	5.8%	14.5%
Age group								
0–18 years	0	0	0	0.3	0	0.1%	0%	0.05%
18–64 years	21.4%	35.2%	24.6%	36.5%	17.4%	21.6%	25.4%	23.5%
65–85 years	57.1%	47.6%	47.2%	50.2%	36.7%	32.1%	46%	37.4%
More than 85 years	14.3%	4.3%	2.6%	4.3%	4.2%	3.3%	7.9%	3.8%
Not specified	7.1%	12.9%	25.6%	8.7%	42%	42.9%	20.6%	35%
Outcome (n, %)								
Serious	100%	98.7%	99%	99.7%	100%	99.5%	98.9%	99.4%
Non serious	0	1.3%	1%	0.3%	0	0.4%	1.1%	0.4%
Not specified	0	0	0	0	0	0.1%	0	0.02%
Region (n, %)								
EEA	0	28.8%	34.4%	10.8%	7.5%	17.7%	19%	16.9%
Non-EEA	100%	71.2%	65.6%	89.2%	92.9%	82.3%	81%	83.1%
Reporter qualification								
HCP	92.9%	55.8%	59.5%	74.6%	34.6%	36.4%	69.3%	44.4%
Non-HCP	7.1%	44.2%	40.5%	25.4%	65.4%	63.6%	30.7%	55.5%

**Figure 2:**
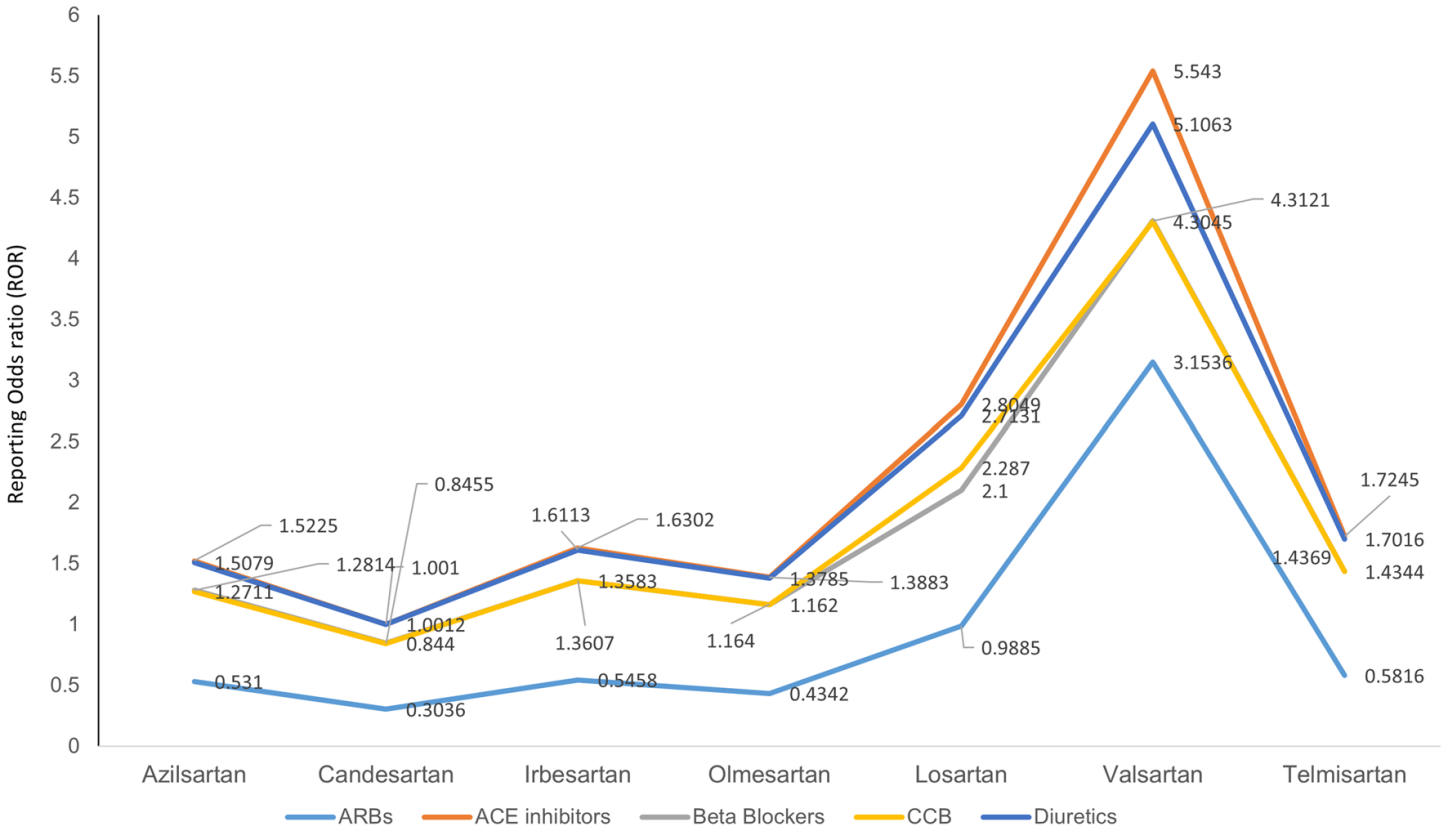
Disproportionality analysis of angiotensin receptor blockers with other antihypertensive drugs.

The study of ARBs neoplasm cases and controls and study with other ARBs showed that neoplasm occurs more often with valsartan (ROR 1.949, 95% CI 1.857–2.046 and ROR 3.153, 95% CI 2.9413–3.3813). Comparative analysis of neoplasm cases where ARBs neoplasm served as reference were studied between ARBs versus ACE inhibitors revealed that neoplasm with ARBs (azilsartan ROR 1.522, 95% CI 0.892–2.598; candesartan ROR 1.00, 95% CI 0.8691–1.153; irbesartan ROR 1.630, 95% CI 1.398–1.900; Olmesartan ROR 1.388, 95% CI 1.226–1.571; losartan ROR 2.804, 95% CI 2.541–3.096; valsartan ROR 5.543 95% CI 5.146–5.97 and telmisartan ROR 1.72, 95% CI 1.476–2.014) was more frequent than with ACE inhibitor. When ARBs were compared to BBs, CCBs, and diuretics, a greater proportion of neoplasm was identified. The ROR for valsartan when compared taking ARBs neoplasm as reference to BBs (ROR 4.312, 95% CI 4.036–4.607), CCBs (ROR 4.304, 95% CI 3.996–4.636) and diuretics (ROR 5.106, 95% CI 4.744–5.495) was more often. The disproportionality analysis is provided in Fig. 2.

With regard to the analysis of fixed dose combinations available in market the occurrence of neoplasm was more frequent for valsartan in combination with amlodipine alone (ROR 1.00; 95% CI 0.886–1.135), valsartan with amlodipine and hydrochlorothiazide (ROR 1.052; 95% CI 0.851–1.301) and valsartan with hydrochlorothiazide alone (ROR 2.028; 95% CI 1.888–2.179) (Table 3).

**Table 3:** Disproportionality analysis of fixed dose combination drugs of ARBs.

Exposure	Total	Cases, n	Non-Cases	ROR (95% CI)	P value
All drugs	4,464,148	2,32,506	42,31,642	Reference	
Candesartan + Hydrochlorothiazide	3710	119	3591	0.613 (0.510 to 0.736)	P < 0.0001
Olmesartan + Amlodipine + Hydrochlorothiazide	849	28	821	0.6230 (0.427 to 0.908)	P = 0.0138
Olmesartan + Hydrochlorothiazide	5768	177	5591	0.591 (0.509 to 0.686)	P < 0.0001
Losartan + Amlodipine besilate	20	0	20	–	
Valsartan + Amlodipine	5163	264	4899	1.0031 (0.886 to 1.135)	P = 0.9608
Valsartan + Amlodipine + Hydrochlorothiazide	1658	90	1568	1.0522 (0.851 to 1.301)	P = 0.6390
Valsartan + Hydrochlorothiazide	8551	829	7722	2.028 (1.8884 to 2.1798)	P < 0.0001
Valsartan + Rosuvastatin	5	0	5	–	

## Discussion and conclusion

This is the first retrospective analysis of neoplasm reports related with ARBs undertaken in EVDAS that covers the entire EEA and Non-EEA. A further comparative analysis of ARBs with other cardiovascular medications and accessible fixed dose combinations is done to underline the significance of this analysis.

ARBs are a class of medications that are used to treat a variety of illnesses, including HTN (high BP) and heart failure. They work by blocking the action of angiotensin II, a hormone that constricts blood vessels and increases BP. In recent years, regulatory agencies have raised concerns about the presence of potentially carcinogenic substances in certain formulations of ARBs. Specifically, nitrosamines and azido compounds have been identified in some ARB products. Nitrosamines are known to have carcinogenic properties and are related with an increased risk of neoplasm. Because of these results, progressive recalls of affected ARB products have been initiated to ensure patient safety. Regulatory agencies and pharmaceutical companies are working together to address this issue, conducting thorough investigations, and implementing measures to prevent the presence of these contaminants in ARB medications.

Our study shows that EVDAS contains 68,522 voluntary reports of neoplasm with ARBs as suspected drug and reported 70% by healthcare professionals. The authors reported highest measure of disproportionality with valsartan. The findings of the study show a substantial association between ARBs and neoplasm, which supports the findings of a recent meta-analysis study published by Sipahi et al.^19^, which found an increased risk of neoplasm in patients treated with ARBs (RR 1.08, 95% CI 1.01–1.15) compared to placebo or comparator drugs. Similarly, the study has also shown association of ARBs especially valsartan with neoplasm (ROR 1.949, 95% CI 1.857–2.046). This is the first study to link ARB drugs with other antihypertensive drugs available in the market. Our study has shown that the combination of valsartan and hydrochlorothiazide has resulted in significant outcomes, with the highest ROR of 2.028 (95% CI 1.888–2.179). This suggests a notable interaction between these drugs when used together in hypertensive patients. Moreover, our research has provided evidence indicating that hydrochlorothiazide is associated with an increased risk of skin cancer^33^. Performing a comparative analysis of ARBs with other cardiovascular drugs and available fixed-dose combinations can provide further insights into their effectiveness and safety profiles.

This type of analysis can help identify potential differences in outcomes, adverse events, or efficacy among different treatment options. It may also help healthcare professionals and patients make informed decisions when selecting appropriate medications for cardiovascular conditions.

The major strengths of the present study are the case validations which supports the high-level evaluation performed in EVDAS and the enormous number of ADR reports that are collected over an extensive time-period in a varied population. One limitation is the high heterogeneity is observed in the reports that affects the quality of reports. An important limitation of this study is related to the fact that drug-induced neoplasm is not obvious. Moreover, the database is globally affected by underreporting, with a study showing that not more than 5–10% of the real incidence of ADRs are reported^34^. Other limitations of spontaneous reporting are lack of time with healthcare professionals to fill the record, lack of understanding of the pharmacovigilance system and lack of information in the ICSRs.

The current evidence on the relationship between ARBs, particularly valsartan, and the risk of neoplasms remains inconclusive and conflicting. Therefore, it is essential for regulatory agencies to continue vigilant monitoring of the safety and efficacy of ARBs to ensure patient well-being. Additionally, comprehensive observational and analytical studies are necessary to further investigate and validate this potential association. Healthcare professionals and patients must stay informed about the latest research findings and regularly consult with medical experts to make well-informed decisions regarding the use of ARBs or any other medications. Ensuring a robust understanding of the risks and benefits is vital for optimizing patient care and treatment outcomes.

## Data Availability

The data is publicly available at “https://www.adrreports.eu/en/search.html”. Anonymized data may be requested from the corresponding author S.R.
